# ASH Meeting 2016: developments in hemostaseology

**DOI:** 10.1007/s12254-017-0339-z

**Published:** 2017-06-01

**Authors:** Clemens Feistritzer, Birgit Mosheimer

**Affiliations:** 1 Internal Medicine V – Hematology and Oncology, Medical University of Innsbruck, Anichstr. 35, 6020 Innsbruck (Tyrol), Austria; 2 Internal Medicine II – Infectiology and Immunology/Tropical Medicine, Rheumatology and Pneumology, Medical University of Innsbruck, Anichstr. 35, 6020 Innsbruck (Tyrol), Austria

**Keywords:** Hemophilia, Gene transfer, Anticoagulation, Cancer, Atrial fibrillation

## Abstract

During the annual meeting of the American Society of Hematology (ASH) in San Diego/California, novel developments in the field of hemostaseology were presented. Alternative treatment strategies besides factor replacement were discussed for patients with hemophilia. One of the highlights of the meeting in this year’s plenary session was the presentation of successful adeno-associated virus mediated gene transfer in patients with hemophilia B leading to sustained elevation of factor IX:C (FIX:c). Other alternative treatment approaches in patients with hemophilia A may include bispecific antibodies mimicking factor VIIIa (FVIIIa) activity or disrupting anticoagulant proteins. Focusing on anticoagulation, data on the use of direct oral anticoagulants (DOACs) in cancer patients with atrial fibrillation as well as treatment of superficial vein thrombosis with rivaroxaban were presented. In this short review, we try to highlight the most important presentations during the ASH meeting 2016.

## Take home message

- Rivaroxaban is noninferior to fondaparinux for treatment of symptomatic superficial vein thrombosis
- In cancer patients with atrial fibrillation, the use of DOACs is safe for stroke prevention
- Gene transfer possibly a potential treatment option in patients with hemophilia B in the near future

## Introduction

The annual meeting of the American Society of Hematology (ASH) was held in San Diego/California from December 3–6, 2016. As every year, a broad spectrum of important developments is hematology—but also in hemostaseology—was discussed by various experts. Highlights in the field of hemophilia included the presentation on adeno-associated virus mediated gene transfer in patients with hemophilia B during this year’s plenary session [1]. Another novel treatment option in patients with hemophilia A was discussed highlighting a humanized bispecific antibody mimicking FVIIIa activity [2]. Relating to anticoagulation, data on the use of direct oral anticoagulants (DOACs) in cancer patients with atrial fibrillation [3] and treatment of superficial vein thrombosis (SVT) with rivaroxaban [4] were presented.

This review will summarize the most relevant topics during the ASH meeting 2016 for the daily clinical work.

## Rivaroxaban vs. fondaparinux in the treatment of superficial vein thrombosis

Management of SVT is based on the risk assessment of developing deep-vein thrombosis and pulmonary embolism (PE). Treatment includes in the low-risk setting topical treatment or nonsteroidal anti-inflammatory drugs (NSAID), in intermediate risk situations fondaparinux 2.5 mg daily for 45 days or intermediate dose low molecular weight heparin (LMWH; for 4–6 weeks), and for high-risk patients therapeutic anticoagulation with vitamin K antagonists (VKA) or DOACs for 3 months (Table 1; [5]). The recommendation for the use of fondaparinux is mainly based on the CALISTO trial [6], a randomized prospective trial including 3002 patients with SVT. The results showed a significant reduction by fondaparinux compared to placebo of the composite endpoint (death from any cause, symptomatic PE or deep vein thrombosis, or extension to the saphenofemoral junction or symptomatic recurrence of SVT; [6]).

**Table 1:** Treatment recommendations for superficial vein thrombosis (*SVT*) of the lower limb (adapted after [5])

SVT – risk stratification	Localization/thrombus length	Treatment
Low risk	Thrombus length <4–5 cm and >3 cm from saphenofemoral/saphenopopliteal junction	Topical or oral NSAID for 8‑12 days
Intermediate risk	Thrombus length >4–5 cm and >3 cm from saphenofemoral/saphenopopliteal junction	Fondaparinux 2.5 mg daily for 45 days or intermediate/therapeutic dose LMWH for 4–6 days or *Rivaroxaban 10 mg*
High risk	Thrombus <3 cm from saphenofemoral/saphenopopliteal junction	Therapeutic anticoagulation as for DVT – VKA/DOAC for 3 month

Recommendations may change depending on the clinical history (e. g., history of previous VTE, active cancer)

In the presented Surprise Trial (ASH# 85; [4]) Beyer-Westendorf et al. compared whether rivaroxaban, an direct oral factor Xa inhibitor, is noninferior to fondaparinux in the prevention of thromboembolic complications in patients with SVT and at least one additional risk factor (older than 65 years, male sex, previous venous thromboembolism, cancer, autoimmune disease, thrombosis of nonvaricose veins). In this open-label randomized, noninferiority phase 3 trial, 472 patients with symptomatic SVT were randomly assigned to the rivaroxaban group (10 mg oral, *n* = 236) or the 2.5 mg fondaparinux group (2.5 mg subcutaneous, *n* = 236). Treatment was given once a day for 45 days. In all, 435 patients were included in the analysis. The primary efficacy outcome occurred in 7 (3%) of 211 patients in the rivaroxaban group and in 4 (2%) of 224 patients in the fondaparinux group (*p* = 0.0025 for noninferiority) at day 45. There were no major bleeds in either group. Consequently, the authors pointed out that rivaroxaban was noninferior to fondaparinux for treatment of SVT in terms of symptomatic deep vein thrombosis or PE, progression or recurrence of SVT, and all-cause mortality [4].

## Direct oral anticoagulants in patients with cancer and atrial fibrillation

The use of DOACs, namely the Xa inhibitors rivaroxaban, apixaban and edoxaban or the IIa antagonist dabigatran, in cancer patients is an ongoing discussion [7]. In cancer-associated venous thromboembolism (VTE) LMWH are still recommended for the initial 3–6 months of treatment due to the lack of randomized, prospective trials. Subsequently, the treatment can be switched to oral anticoagulants of which DOACs and VKA are at least equally effective [8, 9]. However, some clinicians are also reluctant using DOACs in cancer patients with atrial fibrillation (AF) for stroke prevention. Large randomized clinical trials of DOACs compared to warfarin in cancer patients with AF have not been performed. Therefore, different research groups presented their data during the meeting retrospectively analyzing the effectiveness and risk of DOACs vs. warfarin in the real-world population of cancer patients with AF. Shah et al. [3] used MarketScan databases including 532,743 AF patients initiating oral anticoagulant treatment between 2010–2014 and identified 41,036 actively treated cancer patients at the time of anticoagulant initiation. Active cancer patients were defined by the use of chemotherapy, radiation therapy, or cancer surgery within 6 months prior to the start of anticoagulation. DOAC users were matched with warfarin users according, e.g., to age and sex. Study endpoints included ischemic stroke, severe bleeding, or nonsevere bleeding and VTE. A total of number of 6075 cancer patients with AF were treated with DOACs (rivaroxaban *n* = 2808, dabigatran *n* = 2189, and apixaban *n* = 1078) and compared to 10,021 cancer patients on warfarin. The mean age was 74 years, and a mean follow-up time of 1 year.

Patients treated with apixaban had a lower risk of severe bleeding compared to warfarin users (hazard ratio [HR] 0.37, confidence interval [CI] 0.17–0.79, *p* = 0.01). Treatment with rivaroxaban and dabigatran was nonsignificantly different to warfarin for severe bleeding; however, the risk of nonsevere bleeding was lower in the dabigatran group compared to warfarin (HR 0.58, CI 0.41–0.84, *p* = 0.003). The risk of ischemic stroke did not differ; however, each of the DOACs was superior to warfarin in lowering the risk of incident VTE with *p* values <0.0001 [3]. Therefore, it can be concluded that DOACs can be safely used in cancer patients in regard to the management of AF.

## Upcoming treatment options in hemophilia

Prophylactic replacement therapy as standard care for hemophilia in the early 1990s changed health outcomes for hemophilia patients and led to reduction of possible disabling chronic joint damage. However, challenges of prophylactic coagulation factor infusion still remain and include regular and frequent intravenous infusions. Especially in children, but also in older patients, the lack of a reliable intravenous access can lead to decline of adherence of the patients. Finally, development of neutralizing alloantibodies (inhibitors) still occurs in up to 40% of hemophilia patients [10].

New factor proteins with extended half-life can reduce the frequency of intravenous administration. These products are primarily helpful in the treatment of hemophilia B because of the significantly pronounced extension of the half-life of factor IX (FIX) compared to FVIII [11]. Other possible innovations in the hemophilia treatment might include disrupting anticoagulant proteins (Table 2), the according clinical trials are ongoing [12]. Novel treatment options might also include bispecific antibodies to mimic the coagulation function of FVIII or gene transfer for production of endogenous factor protein—these treatment options were highlighted during the ASH meeting 2016.

**Table 2:** Nonfactor replacement strategies – disrupting anticoagulant proteins (adapted after [12])

Product	Mechanism	Clinical trial status
ACE910 – emicizumab	Humanized bispecific antibody, mimics FVIII function	Phase 3
NN7415 – concizumab	mAB against TFPI	Phase I
BAY 1093884	mAB against TFPI	Phase I
ALN-AT3SC –fFitusiran	siRNA targeting antithrombin	Phase 1
BAX 499 (ARC19499) – nonacog gamma	Anti-TFPI Aptamer	Terminated phase 1

## Humanized bispecific antibody mimicking FVIIIA

Emicizumab (ACE910) is a recombinant humanized bispecific antibody that binds to activated FIX and factor X and mimics the cofactor function of FVIII [13]. Earlier in 2016, Shima et al. published a study which enrolled 18 Japanese patients with severe hemophilia A (with or without factor VIII inhibitors) [2]. The study was conducted as a nonrandomized, open-label dose-escalation study. The patients received a dose of 0.3, 1.0, or 3.0 mg per kg of body weight subcutaneous emicizumab weekly for 12 weeks. The end points were safety, pharmacokinetic profiles as well as the annualized bleeding rate. In this study, emicizumab was not associated with serious adverse events or clinically relevant coagulation abnormalities. The median annualized bleeding rates decreased from 32.5 to 4.4 (0.3 mg/kg group), 18.3 to 0.0 (1 mg/kg group), and 15.2 to 0.0 (3 mg/kg group), respectively. The bleeding was independent of the presence of FVIII antibodies. Episodic use of clotting factors to control bleeding was reduced. Once-weekly subcutaneous administration of emicizumab markedly decreased the bleeding rate in patients who have hemophilia A with or without factor VIII inhibitors [2]. Lately, a press release from Roche indicated that during the ongoing HAVEN 1 trial a case of thrombotic microangiopathy occurred when emicizumab was given together with repeated doses of activated prothrombin complex during serious rectal hemorrhage.

## Gene transfer for sustained FIX levels >30% in patients with hemophilia B

During the plenary session Lindsey A. George presented the updated data on the effect of gene transfer in patients with severe hemophilia B [1]. Earlier publications demonstrated long-term expression of FIX (mean FIX:C ~ 5.1%) following AAV8-mediated gene transfer at 2 × 10^12^ vector genomes (vg)/kg in hemophilia B [14]. Achieving higher levels of FIX:C with dose escalation had led to a dose-dependent, capsid-specific immune response that may prevent sustained expression and efficacy [15, 16]. In the presented study the investigational product, SPK-9001, utilizes an efficient AAV capsid (Spark100) with liver-specific tropism. The expression cassette is a codon-optimized, single-stranded transgene encoding FIX Padua, a naturally occurring variant that confers approximately 8‑fold greater specific activity compared to wild-type FIX [17] and therefore could be administered at low doses to achieve hemostatic FIX expression without need for immunosuppression. In the study 7 patients with baseline FIX:C ≤2% were infused with SPK-9001 at a dose of 5 × 10^11^ vg/kg. Steady-state FIX expression is reached by 12 weeks after vector infusion, resulting in a mean FIX:C of 32.3% ± 6.5%. To date, no subjects required immunosuppression or demonstrated evidence of a cytotoxic immune response or liver failure. No patient developed a FIX inhibitor. Only one patient had to be infused with FIX concentrate for a suspected ankle bleed 2 days after vector infusion. The 4 hemophilia patients previously maintained on prophylaxis safely stopped without break-through bleeding.

In summary, preliminary data suggest SPK-9001 safely and consistently produces sustained elevation in FIX:C levels [1].

## Abbreviations

AF: Atrial fibrillation
ASH: American Society of Hematology
CI: Confidence interval
DOAC: Direct oral anticoagulants
FVIII/IX: Factor VIII/IX
HR: Hazard ratio
LMWH: Low molecular weight heparin
NSAID: Nonsteroidal anti-inflammatory drugs
PE: Pulmonary embolism
SVT: Superficial vein thrombosis
VG: Vector genomes
VKA: Vitamin K antagonists
VTE: Venous thromboembolism
